# American Academy of Dental Science

**Published:** 1876-09

**Authors:** 


### ARTICLE II.

### *American Academy of Dental Surgery.*

Reported for the American Journal of Dental Science.

A Special meeting was held on the morning of Tuesday the 25th of July, and a regular meeting of the Academy was held in the afternoon of the same day in Judges Hall, Exposition Grounds, Philadelphia. There was quite an attendance of the medical and dental professions, and much interest was exhibited on the subject for which the meeting convened; and which subject has been agitated by the Academy for months past—that of merging the dental with the medical education.

The morning session was almost entirely devoted to the discussion of the subject, to the exclusion of the reading of papers.

The Chairman of the Executive Committee, Dr. Geo. E. Meigs, presented his report recommending the application of a number of gentlemen to active and corresponding fellowship; they were unanimously elected. Dr. R. Perry, Treasurer, presented his report which was accepted. A number of letters received from Fellows regretting their inability to be present, approving the present action of the Academy, was read by acting Secretary Dr. C. D. Hayward.

VICKSBURG, MISS., June 28th, 1876.

DEAR SIR—

Our State Dental Association will hold its annual session on the 16th of August prox., at Jackson. We confidently expect a full attendance, as the most prominent dentists of the State have expressed a desire to be present and put their shoulders to the wheels of progress and reform. We feel it is indeed high time for us to awake from our slumbers, truly Rip Van Winkle like, at least as to their duration, and contribute our mite, however insignificant, towards the duration of the dental profession, to the same plane socially and professionally occupied by the medical profession. With our profession properly educated and laws enacted prohibiting incompetent persons from practicing dentistry, we shall doubtless receive a cordial recognition from the medical fraternity. There certainly is no good reason why the same respect and importance should not be accorded to the dentist as the Oculist, or any of the specialty. Our Association will make strenuous efforts to induce our next Legislature to enact a law prohibiting incompetent persons or tooth carpenters, as they are facetiously called, from practicing the dental art. Such a law like ballast thrown from the ascending balloon, will lift the profession to a higher and purer level. It is, however, not the dentist only who should be educated, but the people also should be duly enlightened to reap the full benefit of dental surgery.

The young mind cannot too early be made to understand the care and attention his teeth should receive; to this end, judicious suggestions couched in plain language would not be out of place in school, renders that our youth might learn this important lesson as they learn to read. I had hoped to be able to attend the July meeting of the Academy at Philadelphia, but the best laid plans often miss their aim when destiny shapes the end the wrong way, as it has in my case. Regrets were vain, yet I shall nevertheless sensibly feel the great loss that will accrue to me from my absence. Our Northern brethren are cordially invited to participate in the meetings of our Association, and give us the benefit of their greater practical experience, in directing our efforts to successful results.

I herewith send you for your cabinet of curiosities a Liliputian tooth, extracted from the jaw of a negro boy, aged 18 years. It was the first permanent molar superior maxilla, left side; all his other teeth were perfect in size and form, and free from decay and tartar. This tooth is a curious freak of dame nature, and wholly inexplicable on the principle that she makes nothing in vain.

With earnest prayers for the prosperity of the Academy,

I remain my dear sir,

Yours Fraternally,

JNO. D. MILES.

Dr. John Belisario, Sidney, Australia:

"I need not express to you the pleasure it would afford me to be present at the meeting, to say nothing of the great benefit to be derived from rubbing against so many savans. With every hope of success to the Academy, etc."

Dr. W. O. F. Bascome, Hamilton, Bermuda, writes:

"I expected to be with you in Philadelphia on the 25th inst., I am prevented by being obliged to be in attendance at the Court of Her Majesty, to give medical evidence in a case to be tried. I hope with God's help that I will prove a good Fellow, but do not expect too much from me. My English is not good, Spanish is my language. I will try and send from time to time, that which may be acceptable to the Academy and the profession."

Donations were then received with thanks. A back-action plugging instrument and a new substance for filling teeth, from Dr. J. Porter Michael, Paris, France; from John D. Miles, Vicksburg, Miss., a specimen of Liliputian tooth; additions to the collection from Dr. John Belisario, Sidney, Australia; one volume Braithwaits Retrospect of Practical Medicine and Surgery, from Townsend, Adams & Co., New York; three volumes of the Medical Record from Wm. Wood & Co., New York.

Addresses were then made and letters read by and from the following:

The President said the objects and aims of the Academy is to extend the knowledge of dental surgery, to cultivate and develop closer relations professionally among the members at home and abroad, to draw out and mature the latent talent evinced by the young and rising members of the profession, and to secure a higher appreciation of the profession by a progressive improvement of a curriculum of study, having for its foundation—medicine, of which dental surgery is an important branch. In furtherance of these objects, the Academy proposed and encouraged the formation of local organizations in this Country and Europe. Trusting that this our Centennial year will prove a faithful one, I, with pleasure congratulate you upon the occasion of our meeting in Philadelphia, where everything bears the impress of the Centennial ; even the Profession has not escaped the epidemic. I am pleased to see so many of our medical brethren with us here to-day. We feel encouraged with the earnestness of the work done, and proud that the Academy had come to the front. It was their belief that the alienation of their branch of the profession from the parent tree of medicine had been a mistake which could only be retrieved by restoring it to the position to which it properly belonged, and it was their design to advocate and forward with all due influence the institution of dental chairs and professorships in our medical colleges, also the practice of dentistry in the hospitals and the institution of dental practice in the Army and Navy by proper authorization on the part of the Government. He was confident of their ability to succeed in carrying out these intentions for the mutual benefit of all concerned, and felt re-assured by the response received from every quarter.

Prof. Frank H. Hamilton, M. D., New York, said his views are freely given in the address he had the honor to make before the Academy some time since, and which have been published, and which also, I am gratified to know have met your approval. Dentists had nothing to regret. They had done more than could reasonably be expected, and he was convinced that the science and art of dentistry would advance, even though the Academy did not freely accept the plan and methods he had ventured to suggest.

Alfred H. Carroll, M. D., Medical Department, New York University, said he regarded dentistry like all other operative specialties, as a branch of surgery, requiring for its successful practice successful skill and special study, but such study and manipulative dexterity should be superadded to a general knowledge of surgery, not left to limit a mere mechanical routine. Just as one surgeon might concentrate his attention upon the diseases of the eye, ear, or throat, so another might advantageously confine his practice to the teeth, but in either case he should be a surgeon first, a specialist afterward. Ophthalmology, larygology, &c., were fittingly taught from separate chairs in a well appointed curriculum, not in separate colleges, and there could be no reason why dental surgery should be made an exception to the rule. He had endeavored to show how inseparable a general medical education, particularly as regarded nervous disorders, was from skilled dental practice, and he believed that the more the subject was discussed the more intimate would be found the relation between the special and the general. He said that in the English Royal College of Surgeons candidates for diplomas in dental surgery were exempted from examination in midwifery, botany, and one or two other special subjects, manifestly unneeded by them, in lieu of which they were expected to acquire proficiency in the details of their chosen art. Some such substitutive scheme might be adopted in our medical schools, should they establish chairs of dental surgery and mechanical dentistry, conferring special degrees of the practical exigencies of the case. In conclusion, he assured the members of his desire for the success of any measure to bring the means of curing disease under the broad banner of medicine, for said he, " The whole includes every part."

Prof. W. T. Flint, M. D., College of Medicine, New York State, said they were deeply interested in the western part of the State in the subject under discussion. It was their conviction that dentistry should be taught in medical schools, and they had in contemplation the establishment at no distant day, of dental chairs in that College. He believed in the co-education of dental and medical students, and would gladly co-operate in the promotion of the object proposed.

Prof. Joseph Liedy, M. D., University of Pennsylvania, said he knew their faculty felt strongly the importance of dental surgery as a branch of medical education, and had been desirous of having chairs established for the purpose of teaching it in connection with the University. This course had been recommended and urged, but thus far the Board of Trustees had refused, as sufficient influence had not been brought to bear on them by the medical and dental profession. The influence of the academy would do much to promote the object proposed.

A letter was read from Prof. Allen McLane Hamilton, M. D., Long Island College Hospital, indorsing the measure, as he had long recognized the importance of studying the connection of the diseases of the teeth and those of the general nervous system. If there is any way in which I can assist you in your project I am at your service.

Letters were also read from Prof. John M. Carnochan, M. D., New York, encouraging the movement. Prof. Chas. Inslee Pardee, M. D., Medical Department New York University, wrote that he regretted exceedingly that his engagements were such that he was unable to attend the meeting.

A paper by John C. Story, M. D., Dallas, Texas, giving a statement of the condition of the profession in the South-West, and the movement progressing there, was then read and discussed. Several other topics of minor importance came before the meeting, and late in the afternoon, with a vote of thanks to the Commissioners for the use of their room, the Academy adjourned to the fourth Tuesday in September, to meet then in New York.

